# Prospective associations and population impact of sweet beverage intake and type 2 diabetes, and effects of substitutions with alternative beverages

**DOI:** 10.1007/s00125-015-3572-1

**Published:** 2015-05-06

**Authors:** Laura O’Connor, Fumiaki Imamura, Marleen A. H. Lentjes, Kay-Tee Khaw, Nicholas J. Wareham, Nita G. Forouhi

**Affiliations:** Medical Research Council (MRC) Epidemiology Unit, Institute of Metabolic Science, University of Cambridge School of Clinical Medicine, Cambridge Biomedical Campus, PO Box 285, Cambridge, CB2 0QQ UK; Department of Public Health and Primary Care, University of Cambridge, Cambridge, UK

**Keywords:** Adiposity, Coffee, Fruit juice, Population impact, Sweet beverages, Tea, Type 2 diabetes, Water

## Abstract

**Aims/hypothesis:**

This study aimed to evaluate the association of types of sugar-sweetened beverages (SSB) (soft drinks, sweetened-milk beverages, sweetened tea/coffee), artificially sweetened beverages (ASB) and fruit juice with incident type 2 diabetes and determine the effects of substituting non-SSB for SSB and the population-attributable fraction of type 2 diabetes due to total sweet beverages.

**Methods:**

Beverage consumption of 25,639 UK-resident adults without diabetes at baseline (1993–1997) in the European Prospective Investigation into Cancer and Nutrition (EPIC)-Norfolk study was assessed using 7-day food diaries. During 10.8 years of follow-up 847 incident type 2 diabetes cases were verified.

**Results:**

In adjusted Cox regression analyses there were positive associations (HR [95% CI] per serving/day]) for soft drinks 1.21 (1.05, 1.39), sweetened-milk beverages 1.22 (1.05, 1.43) and ASB 1.22 (1.11, 1.33), but not for sweetened tea/coffee 0.98 (0.94, 1.02) or fruit juice 1.01 (0.88, 1.15). Further adjustment for adiposity attenuated the association of ASB, HR 1.06 (0.93, 1.20). There was a positive dose–response relationship with total sweet beverages: HR per 5% energy 1.18 (1.11, 1.26). Substituting ASB for any SSB did not reduce the incidence in analyses accounting for energy intake and adiposity. Substituting one serving/day of water or unsweetened tea/coffee for soft drinks and for sweetened-milk beverages reduced the incidence by 14%–25%. If sweet beverage consumers reduced intake to below 2% energy, 15% of incident diabetes might be prevented.

**Conclusions/interpretation:**

The consumption of soft drinks, sweetened-milk beverages and energy from total sweet beverages was associated with higher type 2 diabetes risk independently of adiposity. Water or unsweetened tea/coffee appear to be suitable alternatives to SSB for diabetes prevention. These findings support the implementation of population-based interventions to reduce SSB consumption and increase the consumption of suitable alternatives.

**Electronic supplementary material:**

The online version of this article (doi:10.1007/s00125-015-3572-1) contains peer-reviewed but unedited supplementary material, which is available to authorised users.

## Introduction

Substantial observational evidence supports a link between consumption of sugar-sweetened beverages (SSB), including soft drinks and cordials, and type 2 diabetes. A meta-analysis of prospective studies reported a relative risk (95% CI) of 1.26 (1.12, 1.41) for highest vs lowest SSB consumption [1]. More recently, in a European study across eight countries, the European Prospective Investigation into Cancer and Nutrition (EPIC)-InterAct Study, we reported a positive association between SSB intake and risk of type 2 diabetes that was independent of several confounding factors, including adiposity [2]. Both null [2, 3] and positive [4, 5] associations have been reported for consumption of artificially sweetened beverages (ASB) and incident type 2 diabetes, although the effect of confounding or of reverse causality by adiposity in this association is possible [6]. Evidence for fruit juice consumption is more limited and inconsistent, with both positive [7, 8] and null associations reported [2, 4, 9]. In addition to these uncertainties, little is known about the association between the consumption of other SSB such as sweetened tea, coffee and milk beverages and type 2 diabetes, nor of an association with per cent contribution to total energy intake (%TEI) consumed as sweet beverages. Moreover, while limiting SSB consumption is recommended by public health agencies [10–12] and taxation of SSB has been considered [13, 14], there is insufficient evidence of what constitutes appropriate replacement beverages to make recommendations [3, 8].

Past research on SSB consumption has predominantly relied on the use of dietary data derived from food frequency questionnaires (FFQs). FFQs are limited in their ability to examine individual beverage types and do not usually link sugar added by participants to beverages such as tea or coffee. Dietary assessment using prospective food diaries can provide the detailed data that overcomes such limitations but this method has not been widely used in epidemiological research.

This study used dietary information from 7 day food diaries and had three linked objectives, to examine: (1) the associations of different types of SSB, such as soft drinks, sweetened-milk beverages and drinks sweetened with sugar post-purchase, ASB and fruit juice with incident type 2 diabetes; (2) whether the contribution of sweet beverages to TEI affects the risk of type 2 diabetes; and (3) the potential effects on type 2 diabetes incidence of substituting alternative beverages for SSB. The population impact of sweet beverage consumption in lowering type 2 diabetes incidence was also evaluated.

## Methods

### Study design

The EPIC-Norfolk study, described in detail previously [15], is a UK population-based cohort of 25,639 men and women aged 40–79 years at baseline in 1993–1997. All volunteers gave written informed consent and attended a health check at their general practitioner’s clinic. The study was approved by the Norfolk Research Ethics Committee.

The current analysis excluded those who did not return a food diary (*n* = 132) and those with: extreme TEI (top and bottom 1% of TEI) (*n* = 256); prevalent or unconfirmed diabetes (*n* = 6); or missing covariates: education level (*n* = 18), family history of diabetes (*n* = 6), alcohol consumption (*n* = 239), smoking status (*n* = 187), self-reported hypertension (*n* = 29) or hypercholesterolaemia (*n* = 65), BMI (*n* = 47) or physical activity level (*n* = 1). A total of 24,653 participants remained for analysis.

### Diabetes cases

Incident type 2 diabetes cases occurring until 31 July 2006 were ascertained using multiple sources: self-report of doctor-diagnosed diabetes from the second health check (3 years post-baseline) or follow-up health and lifestyle questionnaires (18 months and 10 years post-baseline), self-report of diabetes-specific medication in either of the two follow-up questionnaires or medication brought to the follow-up health check. External sources through record linkage were used to verify self-reported type 2 diabetes and identify unreported cases. The date of diagnosis was defined as the earliest date where there was evidence of diabetes from either self-report or from an external source. These included general practice diabetes and local hospital diabetes registers, hospital admissions data with screening for diabetes related admission and Office of National Statistics mortality data with diabetes coding. Participants who self-reported type 2 diabetes that could not be verified with an objective information source were not included as cases (*n* = 5).

### Dietary intake

Baseline dietary intake data were collected using 7 day food diaries [16]. Participants were asked to record everything they ate for 7 consecutive days, covering weekdays and weekend days, with particular attention to amounts and food-preparation methods. Food diaries were collected throughout the year and over a 4 year period, which accounted for seasonal variation in dietary intake at a cohort level. Food and beverage intake data were entered using the programme Data into Nutrients for Epidemiological Research (DINER) [17] and converted into food weights and nutrient intakes using DINERMO [18] (www.epic-norfolk.org.uk/). Intake (g/day) of (1) soft drinks (soft drinks, squashes and juice-based drinks sweetened with sugar), (2) sweetened tea or coffee, (3) sweetened-milk beverages (for example, milkshakes, flavoured milks, hot chocolate), (4) ASB and (5) fruit juice were estimated (further details in Electronic supplementary material [ESM] Table 1). A 1-to-1 g-to-ml conversion was assumed. In these analyses, (1), (2) and (3) are referred to collectively as SSB. TEI (kJ/day) from these sweet beverages was also estimated.

### Covariates

Self-administered questionnaires were used to collect baseline demographic, socioeconomic, lifestyle, physical activity and health characteristics, as described previously [15]. A validated four-point physical activity index was used to categorise participants according to activity level (active, moderately active, moderately inactive, inactive) [19]. Height (cm), weight (kg) and waist circumference (cm) were measured using standardised procedures. Dietary covariates were estimated using the 7-day food diary.

### Statistical analysis

Analyses were performed using Stata (version 13; Stata Corp, College Station, TX, USA). Statistical significance was defined as *p* < 0.05.

Baseline characteristics of the study cohort were described using mean, median or proportion (%). Cox proportional hazards regression was used to estimate HRs and 95% CIs for the prospective association of sweet beverage intake with type 2 diabetes incidence.

Incident type 2 diabetes was examined per intake serving of each sweet beverage. Serving sizes were pragmatically chosen after consideration of median portion sizes of consumers within the study, standard manufacturers’ portion sizes in the UK and guidelines for portion sizes of fruit juice [20]. Assigned serving size varied by beverage: soft drinks and ASB, 336 g/day; sweetened tea or coffee and sweetened-milk beverages, 280 g/day; and fruit juice, 150 g/day. The association was also examined across four intake categories (non-consumers, and consumers categorised by tertiles) for each sweet beverage. The linear trend was examined by modelling the median values for each sweet beverage intake category as a continuous variable.

Age was included as the underlying timescale in Cox models, with entry time defined as age at recruitment and exit time as age at type 2 diabetes incidence, death or censoring at the end of follow-up, whichever came first. The assumption of proportional hazards, checked by including the interaction term between each sweet beverage and age, was not violated.

Analyses were adjusted for age (in addition to as underlying timescale), sex, occupational social class, education level, family history of diabetes, physical activity level, smoking status, alcohol consumption (units/week) and season (date of dietary record dichotomised as winter, summer) and were each mutually adjusted for intake of the other sweet beverages (Model 1). Further adjustment for other food and beverage intake variables (alcoholic beverages, unsweetened tea or coffee, drinking water, fruit, vegetables, processed meat, red meat and fish) little altered the results and were not included in primary analysis. Two subsequent models were constructed, one additionally adjusted for TEI (Model 2) and the second additionally adjusted for TEI, BMI and waist circumference (Model 3), allowing for obesity to be considered as both a mediator and a confounder. Possible interactions between intake of each sweet beverage and age, sex, BMI, waist, physical activity index and smoking status were examined a priori by including interaction terms in the most adjusted models. Interactions were considered significant where *p* < 0.05.

A number of sensitivity analyses were conducted, using Model 3. These included repeating analyses: (1) additionally adjusting for fibre intake to examine the role of nutrient displacement and overall dietary quality; (2) additionally adjusting for saturated fat intake; (3) adjusting for non-sweet-beverage energy intake in place of TEI to reduce the risk of over-adjusting as sweet beverages contribute to TEI; (4) excluding those with prevalent myocardial infarction, stroke and cancer (*n* = 2,332) and separately excluding those with self-reported hypertension or hypercholesterolaemia (*n* = 4,943) to account for possible post-diagnosis changes in diet; (5) excluding those with incomplete food diary records (<7 days) (*n* = 2,219) to assess reporting bias; (6) excluding the top 1% of consumers for each sweet beverage separately to minimise the influence of outliers; (7) excluding those diagnosed with type 2 diabetes within the first 5 years of follow-up (*n* = 237); and (8) excluding those with HbA_1c_ ≥6.5% (48 mmol/mol) at baseline (*n* = 486). Last, BMI, waist circumference and alcohol consumption were adjusted for as categorical covariates rather than continuous variables.

Examining total intake of sweet beverages (soft drinks, sweetened tea or coffee, sweetened-milk beverages, ASB and fruit juice) is also of interest, but summing total g/day was precluded as not all sweet beverages are comparable in composition. Thus, to assess the association of total sweet beverage intake and type 2 diabetes, intake was expressed as %TEI. As ASB do not contain energy, their consumption does not contribute to this variable. A dose–response relationship was examined using a restricted cubic spline with knots at the 25th, 50th and 75th percentiles, in Model 3 without TEI.

The impact of reducing sweet beverage intake on type 2 diabetes incidence was estimated as the per cent population-attributable fraction (PAF) of type 2 diabetes incidence attributable to high %TEI from sweet beverages, under the assumption of causality [21]. Taking into consideration the distribution of intake in the study population and achievable levels of intake, three PAFs with 95% CI were separately estimated (using Model 2), assuming that participants consumed sweet beverages at less than 10%, 5% or 2%TEI, treating %TEI from sweet beverages as a binary variable.

The potential effects of substituting a serving of a non-sugar-sweetened beverage (ASB: 336 g/day; drinking water: 280 g/day; unsweetened tea or coffee: 280 g/day), for a serving of a sweet beverage, were estimated. This was done by examining the difference between regression coefficients for the two beverages, when both beverages were included as continuous terms in a single model (Model 3) mutually adjusted for intake of other sweet beverages and with and without adjustment for TEI [22]; 95% CIs were computed using a variance–covariance matrix for the two beverages.

The association of drinking water and unsweetened tea or coffee per serving with incident type 2 diabetes was examined for the purpose of comparison, using Model 3.

## Results

Nearly all participants consumed at least one sweet beverage (*n* = 24,639 of 24,653) during the 7 days. Soft drinks were the most commonly consumed of the sweet beverages (52.0%) and sweetened tea or coffee contributed most (33%) to the weight of total beverage intake (ESM Table 1). Baseline characteristics of soft drink consumers were broadly similar to those of the total cohort (Table 1). Consumers of sweetened tea or coffee and of sweetened-milk beverages were more likely to be from a lower social class and have generally less healthy diets. The characteristics of ASB consumers were the most different from the total cohort; ASB consumers were younger and more likely to be women, obese and to have reported a family history of diabetes. They also reported being the most physically active and had the lowest TEIs. Fruit juice consumers were of higher social class and had generally healthier diets.

**Table 1:** Baseline characteristics of the total cohort and of consumers of each sweet beverage type: EPIC-Norfolk study (*n* = 24,653)

Characteristic		Consumers only^a^				
	Total cohort (*n* = 24,653)	Soft drinks (*n* = 12,810)	Sweetened tea or coffee (*n* = 12,344)	Sweetened-milk beverages (*n* = 7,428)	ASB (*n* = 5,587)	Fruit juice (*n* = 11,199)
Age (years)	58.7 ± 9.3	58.3 ± 9.2	59 ± 9.2	59.4 ± 9.1	56.3 ± 8.9	58.3 ± 9.2
Sex (% men)	45.3	43.7	54.1	39.4	36.6	39.8
BMI (kg/m^2^)	26.3 ± 3.9	26.2 ± 3.9	25.9 ± 3.6	26.3 ± 3.9	27.2 ± 4.2	26.1 ± 3.8
BMI category						
Underweight (<18.5 kg/m^2^)	0.5	0.5	0.6	0.4	0.3	0.5
Normal weight (≥18.5 < 25 kg/m^2^)	39.1	40.2	42.1	38.9	31.6	41.6
Overweight (≥25 < 30 kg/m^2^)	45.4	44.9	45.2	46.2	47.8	44.6
Obese (≥30 kg/m^2^)	15.0	14.4	12.1	14.5	20.3	13.3
Obese class 1 (≥30 < 35 kg/m^2^)	12.1	11.6	10.4	11.5	14.9	10.7
Obese class 2 and 3 (≥35 kg/m^2^)	2.9	2.9	1.8	2.9	5.3	2.6
Waist circumference (cm)						
Men	95.8 ± 9.8	95.7 ± 9.8	95.0 ± 9.5	95.6 ± 9.4	97.3 ± 9.8	95.4 ± 9.5
Women	82.0 ± 10.8	81.7 ± 10.6	81.2 ± 10.2	82.0 ± 10.7	83.5 ± 11.5	81.3 ± 10.5
Myocardial infarction^b^	3.1	2.9	3.2	3.3	2.9	2.7
Stroke^b^	1.3	1.2	1.3	1.4	1.1	1.1
Cancer^b^	5.5	5.8	5.2	5.6	5.5	5.8
Hypertension^b^	14.2	14.0	13.3	15.2	14.2	13.9
Hypercholesterolaemia^b^	8.2	8.3	7.9	8.4	7.9	8.3
Family history of diabetes^b^	12.9	13.1	11.9	13.8	15.6	13.0
Smoker (current)	11.6	9.9	14.4	7.7	8.8	8.0
Alcohol consumption (units/week)	3.5 (1, 10)	4 (1.5, 10)	3.5 (1, 9.5)	2.5 (1, 7)	4.0 (1.5, 10)	4.5 (1.5, 10)
Physical activity index (active)	18.1	18.9	19.0	18.7	19.2	18.4
Education level (achieved university degree or equivalent)	13.0	13.3	11.5	10.8	12.2	17.4
Social class (professional/managerial)	43.6	44.4	40.4	39.5	44.4	50.9
Dietary intake						
Total energy (kJ/day)	8,159 ± 2,125	8,389 ± 2,071	8,632 ± 2,113	8,213 ± 2,063	7,899 ± 2,046	8,263 ± 2,025
Fibre (non-starch polysaccharide) (g/day)	14.2 (11.3, 17.9)	14.2 (11.3, 17.7)	13.9 (11.0, 17.4)	14.3 (11.4, 17.9)	14.7 (11.7, 18.3)	14.8 (11.9, 18.4)
Fruit (g/day)	149 (76, 239)	152 (83, 239)	132 (64, 215)	153 (86, 238)	167 (93, 261)	167 (98, 255)
Vegetable (g/day)	140 (99, 190)	141 (102, 190)	134 (96, 182)	138 (99, 184)	146 (104, 195)	148 (109, 198)
Fish (g/day)	22 (8, 39)	23 (11, 40)	22 (9, 39)	23 (11, 40)	22 (9, 40)	24 (11, 41)
Unprocessed red meat (g/day)	29 (12, 50)	30 (13, 49)	32 (15, 52)	28 (12, 47)	29 (12, 48)	29 (11, 48)
Processed meat (g/day)	18 (7, 32)	19 (9, 32)	20 (9, 33)	18 (8, 31)	18 (8, 32)	18 (7, 30)

Data are mean ± SD, % or median (interquartile range), unless otherwise indicated

^a^Consumer groups are not mutually exclusive

^b^Self-reported at baseline

During 248,264 person-years, 847 type 2 diabetes cases were identified. When adjusting for potential confounders and TEI (Model 2) a higher type 2 diabetes incidence was observed per serving of: soft drinks, HR (95% CI) 1.21 (1.05, 1.39); sweetened-milk beverages, 1.22 (1.05, 1.43); and ASB, 1.22 (1.11, 1.33) (Table 2). Further adjustment for BMI and waist circumference attenuated the association for ASB, 1.06 (0.93, 1.20) making it non-significant, while the significant associations of soft drinks, 1.14 (1.01, 1.32) and sweetened-milk beverages, 1.27 (1.09, 1.48), were retained. Estimates using the categorical measures were largely similar to those using continuous estimates, except for intake of soft drinks, which were attenuated and became non-significant after adjustment for adiposity (highest intake compared with non-consumers, 1.13 (0.94, 1.36; *p* linear trend, 0.306), possibly because the smaller sample size reduced the power to detect differences. Intake of sweetened tea or coffee and fruit juice were not significantly associated with type 2 diabetes incidence using either the continuous or the categorical estimates. No significant interactions with age (*p* ≥ 0.483), sex (*p* ≥ 0.090), BMI (*p* ≥ 0.424), waist (*p* ≥ 0.182), physical activity level (*p* ≥ 0.099) or smoking status (*p* ≥ 0.274) were evident.

**Table 2:** Prospective association of sweet beverage consumption and type 2 diabetes, HR (95% CI): the EPIC-Norfolk study (*n* = 24,653)

Beverage/model	Per serving	Non-consumers	Consumers			
			Tertile 1	Tertile 2	Tertile 3	*p* linear trend
Soft drinks: range (median) intake (g/day)	336 g/day	0 (0)	>0–49 (35)	50–139 (83)	140–3,172 (234)	
Cases/participants	847/24,653	418/11,843	130/4,297	135/4,243	164/4,270	
Crude	1.19 (1.03, 1.37)	1	0.86 (0.71, 1.05)	0.91 (0.75, 1.11)	1.14 (0.95, 1.36)	0.419
Adjusted model (Model 1)^a^	1.18 (1.03, 1.35)	1	0.92 (0.76, 1.12)	0.97 (0.80, 1.18)	1.17 (0.97, 1.40)	0.202
+TEI (Model 2)	1.21 (1.05, 1.39)	1	0.94 (0.77, 1.14)	0.99 (0.81, 1.21)	1.21 (1.00, 1.45)	0.104
+BMI and waist circumference (Model 3)	1.14 (1.01, 1.32)	1	0.97 (0.80, 1.18)	0.98 (0.80, 1.19)	1.13 (0.94, 1.36)	0.306
Sweetened tea or coffee: range (median) intake (g/day)	280 g/day	0 (0)	1–232 (80)	233–881 (517)	882–5,096 (1,275)	
Cases/participants	847/24,653	394/12,309	149/4,115	166/4,116	138/4,113	
Crude	1.01 (0.98, 1.05)	1	1.08 (0.89, 1.30)	1.23 (1.03, 1.47)	1.05 (0.86, 1.27)	0.190
Adjusted model (Model 1)^a^	0.97 (0.93, 1.01)	1	1.08 (0.90, 1.31)	1.13 (0.94, 1.36)	0.86 (0.70, 1.06)	0.528
+TEI (Model 2)	0.98 (0.94, 1.02)	1	1.11 (0.92, 1.34)	1.16 (0.97, 1.40)	0.91 (0.74, 1.13)	0.963
+BMI and waist circumference (Model 3)	1.03 (0.99, 1.07)	1	1.21 (1.00, 1.46)	1.35 (1.12, 1.63)	1.19 (0.97, 1.47)	0.009
Sweetened-milk beverages: range (median) intake (g/day)	280 g/day	0 (0)	1–74 (40)	75–210 (129)	211–2,653 (280)	
Cases/participants	847/24,653	549/17,225	87/2,485	102/2,467	109/2,476	
Crude	1.20 (1.03, 1.40)	1	1.10 (0.88, 1.38)	1.21 (0.98, 1.50)	1.25 (1.02, 1.54)	0.009
Adjusted model (Model 1)^a^	1.19 (1.02, 1.40)	1	1.19 (0.95, 1.50)	1.26 (1.02, 1.56)	1.25 (1.01, 1.54)	0.006
+TEI (Model 2)	1.22 (1.05, 1.43)	1	1.20 (0.96, 1.51)	1.28 (1.03, 1.58)	1.29 (1.04, 1.59)	0.003
+BMI and waist circumference (Model 3)	1.27 (1.09, 1.48)	1	1.20 (0.95, 1.51)	1.32 (1.07, 1.64)	1.35 (1.10, 1.67)	<0.001
ASB	336 g/day	0 (0)	1–59 (36)	60–168 (99)	169–5,848 (290)	
Cases/participants	847/24,653	634/19,066	58/1,863	70/1,863	85/1,861	
Crude	1.26 (1.15, 1.37)	1	1.04 (0.79, 1.36)	1.30 (1.01, 1.66)	1.70 (1.35,2.14)	<0.001
Adjusted model (Model 1)^a^	1.22 (1.11, 1.33)	1	1.06 (0.81, 1.39)	1.33 (1.04, 1.71)	1.69 (1.34,2.13)	<0.001
+TEI (Model 2)	1.22 (1.11, 1.33)	1	1.06 (0.81, 1.39)	1.33 (1.04, 1.71)	1.67 (1.33,2.11)	<0.001
+BMI and waist circumference (Model 3)	1.06 (0.93, 1.20)	1	0.97 (0.74, 1.27)	1.16 (0.90, 1.49)	1.17 (0.93, 1.48)	0.124
Fruit juice: range (median) intake (g/day)	150 g/day	0 (0)	1–40 (21)	41–122 (77)	123–1,372 (175)	
Cases/participants	847/24,653	524/13,454	97/3,849	109/3,618	117/3,732	
Crude	0.92 (0.80, 1.05)	1	0.65 (0.53, 0.81)	0.78 (0.63, 0.96)	0.81 (0.66, 0.99)	0.003
Adjusted model (Model 1)	0.99 (0.87, 1.13)	1	0.75 (0.60, 0.93)	0.86 (0.70, 1.07)	0.91 (0.74, 1.12)	0.172
+TEI (Model 2)	1.01 (0.88, 1.15)	1	0.76 (0.61, 0.95)	0.88 (0.71, 1.09)	0.93 (0.76, 1.15)	0.259
+BMI and waist circumference (Model 3)	1.04 (0.92, 1.19)	1	0.81 (0.65, 1.01)	0.94 (0.76, 1.16)	0.99 (0.80, 1.22)	0.678

*p* linear trend was estimated by including as the exposure the median of each category as a continuous variable

^a^Adjusted for age, sex, social class (professional, managerial, skilled, semi-skilled, unskilled), education level (no qualification, O level/GCSE [aged 16], A level [aged 18], university degree or equivalent, higher), family history of diabetes (no, yes), physical activity level (active, moderately active, moderately inactive, inactive), smoking status (current, former, never), alcohol consumption, season (winter, summer), mutual adjustment for intake of other sweet beverages

Sensitivity analyses had no substantial impact on the effect estimates (ESM Table 2).

**Fig. 1:**
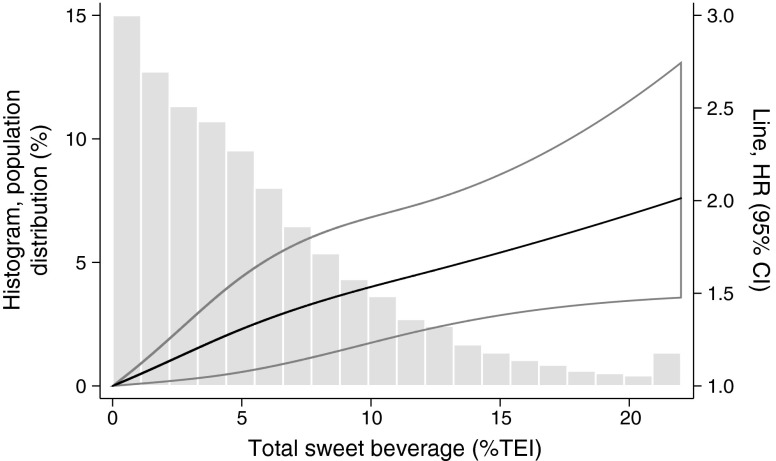
The association of total sweet beverage consumption (% of total energy intake, %TEI) and type 2 diabetes: the EPIC-Norfolk study (*n* = 24,653). %TEI was truncated at 22%. The spline-regression model adjusted for age, sex, social class (professional, managerial, skilled, semi-skilled, unskilled), education level (no qualification, O level/GCSE [aged 16], A level [aged 18], university degree or equivalent, higher), family history of diabetes (no, yes), physical activity level (active, moderately active, moderately inactive, inactive), smoking status (current, former, never), alcohol consumption, season (winter, summer), BMI and waist circumference. As ASB do not contain energy, their consumption does not contribute to this %TEI variable

There was a positive linear association (*p* linear association < 0.001, *p* non-linear association = 0.28) between total sweet beverage intake (%TEI) and incident type 2 diabetes, which was significant at intake above 1%TEI. Each 5% higher intake was associated with an 18% (95% CI 11%, 26%) higher incidence of type 2 diabetes (Fig. 1). Further adjustment for TEI did not change the effect estimate.

The PAF of type 2 diabetes incidence was estimated as 3% (95% CI 1%, 7%) if all adults (aged 40–79 years) reduced sweet beverage intake to below 10%TEI. If intake was reduced to below 5%TEI, the PAF was estimated as 7% (1%, 13%) and if intake was reduced to below 2%TEI, the PAF was estimated as 15% (3%, 25%).

Substituting ASB for soft drinks or sweetened-milk beverages was not associated with type 2 diabetes incidence significantly, HR 0.93 (95% CI 0.75, 1.11) and 0.84 (0.67, 1.00), respectively (Table 3). Substituting drinking water for soft drinks and for sweetened-milk beverages was estimated to reduce type 2 diabetes incidence, 0.86 (0.74, 0.99) and 0.80 (0.67, 0.94), respectively. Substituting unsweetened tea or coffee for soft drinks and for sweetened-milk beverages was also estimated to reduce type 2 diabetes incidence, 0.86 (0.73, 0.99) and 0.75 (0.63, 0.86), respectively. Consuming unsweetened tea or coffee in place of sweetened tea or coffee was also estimated to reduce type 2 diabetes incidence, 0.96 (0.92, 0.99). Results without adjustment for TEI were similar (ESM Table 3). Substituting equivalent serving sizes of non-caloric beverages for sweet beverages rather than using beverage-specific serving sizes did not materially change the results (data not shown).

**Table 3:** The estimated effect of substituting a serving of non-sugar-sweetened beverage for a sweet beverage on incident type 2 diabetes, HR (95% CI): the EPIC-Norfolk study (*n* = 24,653)

SSB	HR (95% CI) for the effect of substituting^a^ a serving of:		
	ASB (336 g/day)	Drinking water (280 g/day)	Unsweetened tea or coffee (280 g/day)
Adjusted model^b^			
Soft drinks (336 g)	0.93 (0.75, 1.11)	0.86 (0.74, 0.99)	0.86 (0.73, 0.99)
Sweetened tea or coffee (280 g)	1.03 (0.89, 1.18)	0.99 (0.91, 1.08)	0.96 (0.92, 0.99)
Sweetened-milk beverages (280 g)	0.84 (0.67, 1.00)	0.80 (0.67, 0.94)	0.75 (0.63, 0.86)
ASB (336 g)	–	0.96 (0.81, 1.11)	0.89 (0.76, 1.02)
Fruit juice (150 g)	1.01 (0.82,1.20)	0.98 (0.82, 1.13)	0.90 (0.78, 1.03)

^a^Estimates for the effect of substitution were calculated as the difference in regression coefficients between the two beverages, when both beverages (the sweet beverage and the non-caloric replacement beverage) were included in a single adjusted model as continuous variables

^b^Adjusted for age, sex, social class (professional, managerial, skilled, semi-skilled, unskilled), education level (no qualification, O level/GCSE [aged 16], A level [aged 18], university degree or equivalent, higher), family history of diabetes (no, yes), physical activity level (active, moderately active, moderately inactive, inactive), smoking status (current, former, never), alcohol consumption, season (winter, summer), total energy intake, BMI and waist circumference

Drinking water intake was not significantly associated with type 2 diabetes, 1.02 (0.95, 1.11) per serving (280 g/day), while unsweetened tea or coffee intake was inversely associated, 0.92 (0.89, 0.96), per serving (280 g/day).

## Discussion

The current findings of an independent positive association of soft drink intake and null associations of ASB and fruit juice intake with incident type 2 diabetes using detailed dietary information from prospective 7 day food diaries further supports the findings from previous studies that used dietary information from retrospective FFQs. This study additionally reports several novel findings. This is the first report of a positive association of sweetened-milk beverages with type 2 diabetes. We also examined the association between the contribution of %TEI from total sweet beverages and type 2 diabetes, finding that each 5% increase in contribution to TEI was associated with an 18% increase in type 2 diabetes incidence. Furthermore, the population impact of sweet beverage consumption on type 2 diabetes has not been previously evaluated; we estimated that 3–15% of incident diabetes cases might be prevented if consumers of sweet beverages reduced their intake to below a range between 10% and 2%TEI. We also report that replacing soft drinks and sweetened-milk beverages with ASB did not reduce type 2 diabetes incidence, but drinking water or unsweetened tea or coffee as alternatives to soft drinks and sweetened-milk beverages lowered the incidence of type 2 diabetes significantly. These novel findings are of clinical and public health relevance.

SSB, a group comprising soft drinks and cordials, have been consistently reported to be associated with increased risk of type 2 diabetes [1] and the association with type 2 diabetes has been shown to be independent of BMI [2, 4]. In the current study, the category ‘soft drinks’ is largely commensurate with SSB as defined in other studies [1, 2, 4], and the current findings are in keeping with other publications, with an increased risk of type 2 diabetes per serving of soft drinks, independent of adiposity.

There is accumulated evidence, including a meta-analysis [23], to suggest that both tea and coffee are inversely associated with incident diabetes. Yet to our knowledge, this is the first epidemiological study to distinguish between sweetened and unsweetened tea or coffee. In the current study, unsweetened tea or coffee was inversely associated with incident diabetes but sweetened tea or coffee overall had a null association. This is also the first study to consider sweetened-milk beverages in longitudinal epidemiological research, reporting a positive association between consumption of sweetened-milk beverages and type 2 diabetes. As added sugar contributes about half of the total sugar content of beverages such as milkshakes and flavoured milks [24], this association is unsurprising. This finding is of concern because flavoured milk is now recognised as a major contributor to added sugar intake in the USA [25]. In addition to our study of older adults, further research, particularly in other age groups, is necessary before any firm conclusions can be drawn.

Although a strong positive association of ASB consumption and incident type 2 diabetes was found, after adjusting for adiposity this became null. Findings from other studies are inconsistent, with both positive [4, 5] and null [2, 26] associations reported. The positive association of ASB and type 2 diabetes may be an artefact of reverse causality where those who are overweight or obese and at higher risk of chronic disease consume a higher amount of ASB than those at lower risk [6]. This was supported in the current analyses in which ASB consumers were more likely to be classified as obese. An earlier study that accounted for pre-enrolment weight change [3] reported a null association between ASB consumption and type 2 diabetes, lending support to the confounding effect of adiposity on the association of ASB consumption and type 2 diabetes incidence.

In the current study, there was a null association of fruit juice intake with risk of type 2 diabetes. This is consistent with a recent meta-analysis of four studies that reported a null association of 100% fruit juice with type 2 diabetes [27].

The incidence of type 2 diabetes was 18% higher per 5% higher TEI from sweet beverages. This was significantly higher at contributions as low as 1%. While not directly comparable, a study in the USA reported an association between the contribution of added sugar above 10% of TEI and higher mortality from cardiovascular disease [28]. Public health recommendations are to restrict the contribution to energy from sugars [29]. As non-alcoholic beverages are a major source of sugars in the UK and USA [30, 31], our findings support these recommendations.

SSB have been considered contributing factors to the diabetes epidemic [6], and while the public health message is to reduce consumption, alternative beverages must be suggested to achieve this. However, there is a paucity of evidence for suitable alternatives in the context of disease risk, other than for weight loss. Findings from two cohorts of US health professionals generally supported the evidence for risk reduction for type 2 diabetes by replacement of SSB [3, 8]. Risk reduction in the first cohort was reported for replacement of SSB with coffee [3]; in the second, risk reduction was reported for replacement of SSB and fruit juice with beverages including plain water, milk, ASB and coffee, but not tea [8]. Unlike earlier studies, the current study examined the effect of substituting each sweet beverage with a non-sugar-sweetened alternative, and sweetened and unsweetened tea or coffee were differentiated. The results of these analyses give practical suggestions for alternatives to SSB and highlight the benefits of substituting SSB with water and unsweetened tea or coffee over ASB. Substituting SSB for ASB is potentially beneficial for reducing risk of type 2 diabetes through reduced TEI and weight. Our study could not evaluate this due to confounding bias in models with ASB without adjustment for adiposity measures.

To the best of the authors’ knowledge, our estimates of the population impact of sweet beverage consumption in reducing type 2 diabetes incidence are the first reported. Our findings are of considerable public health relevance, showing that 3%–15% of type 2 diabetes cases might be prevented under different intake assumptions, as proposed.

The potential biological mechanisms by which SSB may increase risk of type 2 diabetes were not investigated in this study. They are comprehensively reviewed elsewhere [6]. SSB may contribute to type 2 diabetes risk via both their effects on adiposity, where energy intake in liquid form is not fully compensated, promoting weight gain via the glycaemic effect of consuming large amounts of rapidly absorbable sugars, and the metabolic effects of fructose. In the current study, the association of sweetened-milk-beverage intake and type 2 diabetes incidence did not appear to be mediated by adiposity. We postulate that this lack of mediation could be due to possible beneficial effects on satiety from milk protein [32, 33], but our study was not designed to investigate this further.

The limitations of this study warrant consideration. Although food diary data are detailed and more comprehensive than FFQ data, their greater respondent burden could lead to possible reporting bias. Dietary intake was assessed at baseline only and change in sweet beverage consumption over time was not accounted for. As current nationally representative UK adult intakes (g/day) of sweet beverages are 30% higher [34] than in this study, it is likely that the PAF modelled here was underestimated and that greater benefit is possible. Our findings for soft drinks were, however, comparable in magnitude with those of studies that have accounted for dietary change [3, 26]. Although adjustments were made for a wide range of potential confounders, residual confounding is possible due to imprecisely or unmeasured characteristics. As the study population is predominantly white European, these findings may not be generalisable to other populations.

The strengths of this study include the use of prospective food diary data. This allowed for the differentiation of sweet beverage intake into distinct beverage groups, diminishing the chance of masking divergent associations. This also allowed for the inclusion of beverages not typically previously examined in SSB research, including tea or coffee with sugar added by the consumer and sweetened-milk beverages such as flavoured milks and milkshakes. Incident type 2 diabetes cases were ascertained and verified using data internal and external to the study through record linkage and were not dependent on follow-up within the study. Although this method of ascertainment may lead to misclassification of participants with undiagnosed diabetes as non-diabetic individuals, analyses excluding participants with baseline HbA_1c_ measurements ≥ 6.5% (48 mmol/mol) did not affect the conclusions.

In summary, consumption of SSB such as soft drinks and sweetened-milk beverages was associated with higher type 2 diabetes risk independently of socio-demographic, lifestyle and dietary factors, as well as adiposity in this large prospective study. Our findings suggest that reducing consumption of sweet beverages, in particular soft drinks and sweetened-milk beverages, and promoting drinking water and unsweetened tea or coffee as alternatives may help curb the escalating diabetes epidemic. In light of the consistency of past evidence, together with the new evidence generated by this work, it is now timely and appropriate to consider population-based interventions to reduce SSB consumption and increase the consumption of suitable alternative beverages.

## Electronic supplementary material

ESM Table 1 (PDF 47 kb)

ESM Table 2 (PDF 59 kb)

ESM Table 3 (PDF 45 kb)

## Abbreviations

%TEI: Per cent contribution to total energy intake
ASB: Artificially sweetened beverages
EPIC: European Prospective Investigation into Cancer and Nutrition
FFQ: Food frequency questionnaire
MRC: Medical Research Council
PAF: Population-attributable fraction
SSB: Sugar-sweetened beverages
TEI: Total energy intake
