# Supplementary material for: Prospective associations and population impact of sweet beverage intake and type 2 diabetes, and effects of substitutions with alternative beverages
Source: Diabetologia. 2015 May 6;58(7):1474–83. doi: 10.1007/s00125-015-3572-1 (PMC4473082; doi:10.1007/s00125-015-3572-1)
Supplement: Supplementary file 1 — (PDF 47 kb) [file 125_2015_3572_MOESM1_ESM.pdf]

ESM Table 1. Sweet beverage consumption in the EPIC-Norfolk study (n=24653): beverage category descriptions, consumers, intakes and contribution to the weight of total beverage intake[a]

Beverages	Description	Consumers, n (%)	Intakes in consumers only, g/d	Contribution to total weight of beverage intake, %	Consumer of other sweet beverages, %				
					Soft drinks	Sweet tea/coffee	Sweet milk beverages	ASB	Fruit juice
Sugar-sweetened beverages									
Soft drinks	Soft drinks, sports/energy drinks and squashes sweetened with sugar and juice based beverages	12810 (52)	83 (41,176)[b]	5 (2,11)[b]	100	53.5	31.9	23.6	52
Sweetened tea/coffee	Instant and filtered, caffeinated and decaffeinated tea and coffee where table sugar has been added[c]	12344 (50)	516 (147,1074)	33 (9,72)	55.5	100	31.2	17.6	43.5
Sweetened-milk beverages	Milk shakes, flavoured milks, hot chocolates and other hot and cold sweetened-milk based beverages	7428 (30)	157 (56,257)	9 (4,15)	55.0	51.8	100	23.7	48.4
Artificially-sweetened beverages (ASB)	Diet/sugar-free soft drinks, sports/energy drinks and squashes	5587 (23)	98 (47,217)	6 (3,13)	54.1	38.8	31.5	100	49.7
Fruit juice	100% fruit juice and nectars[d]	11199 (45)	75 (35,148)	4 (2,9)	59.5	47.9	32.1	24.8	100

[a] Total beverages include all sweet beverages listed above, water, unsweetened tea or coffee, stock based drinks (e.g. Bovril) and all alcoholic beverages. Milk was not included as milk consumed as a beverage could not be differentiated from milk consumed as a food e.g. with cereal.
[b] Median (IQR, inter-quartile range), all such values.
[c] Tea or coffee sweetened with artificial sweeteners and/or alternative sweeteners such as honey were not identified and were thus included as unsweetened tea or coffee.
[d] Includes juice recorded without further specification.
