# Supplementary material for: Prospective associations and population impact of sweet beverage intake and type 2 diabetes, and effects of substitutions with alternative beverages
Source: Diabetologia. 2015 May 6;58(7):1474–83. doi: 10.1007/s00125-015-3572-1 (PMC4473082; doi:10.1007/s00125-015-3572-1)
Supplement: Supplementary file 2 — (PDF 59 kb) [file 125_2015_3572_MOESM2_ESM.pdf]

ESM Table 2. Sensitivity analyses of the prospective association of sweet beverage consumption (per serving) and type 2 diabetes: the EPIC-Norfolk Study (n=24653)

	Hazard ratio (95% confidence interval) per 1 serving/day of each type of beverages				
	Soft drinks (336g/d)	Sweetened tea or coffee (280g/d)	Sweetened-milk beverages (280g/d)	Artificially-sweetened beverages (336g/d)	Fruit juice (150g/d)
Adjusted model[a]	1.14 (1.01,1.32)	1.03 (0.99,1.07)	1.27 (1.09,1.48)	1.06 (0.93,1.20)	1.04 (0.92,1.19)
+ fibre (non-starch polysaccharides) intake	1.13 (1.01,1.30)	1.01 (0.97,1.05)	1.26 (1.08,1.47)	1.09 (0.95,1.24)	1.05 (0.92,1.19)
+ saturated fat intake	1.15 (0.99,1.33)	1.02 (0.98,1.06)	1.28 (1.10,1.49)	1.06 (0.93,1.22)	1.04 (0.91,1.18)
Non-sweet beverage energy intake in place of total energy intake	1.13 (1.01,1.31)	1.03 (0.99,1.07)	1.26 (1.08,1.46)	1.06 (0.93,1.21)	1.04 (0.91,1.18)
Excluding those with chronic disease at baseline (*n*=22845)	1.13 (0.97,1.33)	1.04 (0.99,1.08)	1.30 (1.11,1.53)	1.09 (0.96,1.24)	1.02 (0.89,1.18)
Excluding those with self-reported hypertension or hypercholesterolaemia at baseline (*n*=20101)	1.21 (1.02,1.42)	1.04 (0.99,1.08)	1.28 (1.06,1.53)	1.13 (0.93,1.38)	1.09 (0.94,1.28)
Excluding those with incomplete food diary records (<7 days) (*n*=22434)	1.12 (0.94,1.32)	1.03 (0.99,1.07)	1.26 (1.07,1.48)	1.02 (0.86,1.20)	1.09 (0.94,1.25)
Excluding the top 1% of consumers for each sweet beverage separately	1.17 (0.95,1.45)	1.03 (0.99,1.08)	1.45 (1.21,1.73)	1.06 (0.93,1.21)	1.02 (0.87,1.20)
Excluding those diagnosed with type 2 diabetes within the first 5 years of follow-up (*n*=24416)	1.14 (0.96,1.26)	1.02 (0.97,1.06)	1.33 (1.12,1.58)	1.03 (0.88,1.22)	1.07 (0.92,1.25)
Excluding those with Haemoglobin A1c ≥6.5% at baseline (n=24167)[b]	1.20 (1.03,1.40)	1.04 (1.00,1.09)	1.28 (1.09,1.51)	1.09 (0.95,1.26)	0.96 (0.83,1.12)
Adjusting for BMI, waist circumference and alcohol consumption as categorical rather than continuous covariates	1.16 (1.00,1.35)	1.03 (0.99,1.07)	1.27 (1.09,1.48)	1.09 (0.97,1.23)	1.03 (0.90,1.17)

[a]Adjusted for age, sex, social class (professional, managerial, skilled, semi-skilled, unskilled), education level (no qualification, O level, A level, degree, higher), family history of diabetes (no, yes), physical activity level (active, moderately active, moderately inactive, inactive), smoking status (current, former, never), alcohol consumption, season (winter, summer), mutual adjustment for intake of other sweet beverages, total energy intake, BMI, waist circumference.

[b] Haemoglobin A1c measurements were only available for n=10976 as funding for measurement only became available in 1995. No significant interaction was noted on the risk of incident diabetes of any sweet beverage intake and availability of Haemoglobin A1c measurement.
