# Supplementary material for: Prospective associations and population impact of sweet beverage intake and type 2 diabetes, and effects of substitutions with alternative beverages
Source: Diabetologia. 2015 May 6;58(7):1474–83. doi: 10.1007/s00125-015-3572-1 (PMC4473082; doi:10.1007/s00125-015-3572-1)
Supplement: Supplementary file 3 — (PDF 45 kb) [file 125_2015_3572_MOESM3_ESM.pdf]

ESM **Table 3.** The effect of substituting a serving of non-sugar sweetened beverages for sweet beverages on incident type 2 diabetes, without adjustment for energy intake: the EPIC-Norfolk Study (*n*=24653)

	Hazard ratio (95% confidence interval) for the effect of substituting[a] a serving of:		
	Artificially-sweetened beverages (336g/d)	Drinking water (280g/d)	Unsweetened tea or coffee (280g/d)
Adjusted model[b]			
Soft drinks (336g)	0.95 (0.76,1.13)	0.87 (0.74,0.99)	0.87 (0.75,0.99)
Sweetened tea or coffee (280g)	1.04 (0.90,1.18)	1.00 (0.92,1.08)	0.96 (0.93,1.00)
Sweetened milk beverages (280g)	0.85 (0.68,1.02)	0.82 (0.68,0.96)	0.77 (0.65,0.88)
Artificially-sweetened beverage (336g)	n/a	0.97 (0.82,1.12)	0.90 (0.77,1.03)
Fruit juice (150g)	1.02 (0.82,1.20)	0.98 (0.83,1.14)	0.91 (0.79,1.04)

[a] Estimates for the effect of substitution were calculated by including both beverages (the sweet beverage and the non-caloric replacement beverage) in a model as continuous variables. The difference in the coefficients was used to calculate the hazard ratio and 95% confidence interval for the substitution effect.

[b] Adjusted for age, sex, social class (professional, managerial, skilled, semi-skilled, unskilled), education level (no qualification, O level, A level, degree, higher), family history of diabetes (no, yes), physical activity level (active, moderately active, moderately inactive, inactive), smoking status (current, former, never), alcohol consumption, season (winter, summer), body mass index, waist circumference
